# Discontinuing the recommendation of hip precautions does not increase the risk of early dislocation after primary total hip arthroplasty using 36-mm heads: a population-based study from the Danish Hip Arthroplasty Register

**DOI:** 10.2340/17453674.2024.41168

**Published:** 2024-07-18

**Authors:** Afrim ILJAZI, Michala Skovlund SØRENSEN, Matilde WINTHER-JENSEN, Søren OVERGAARD, Michael Mørk PETERSEN

**Affiliations:** 1 Department of Orthopedic Surgery, Copenhagen University Hospital Rigshospitalet; 2 Department of Orthopedic Surgery, Zealand University Hospital; 3 Department of Data, Biostatistics and Pharmacoepidemiology, Centre for Clinical Research and Prevention, Copenhagen University Hospital Bispebjerg-Frederiksberg; 4 Department of Orthopedic Surgery and Traumatology, Copenhagen University Hospital Bispebjerg-Frederiksberg; 5 Department of Clinical Medicine, Faculty of Health Science, University of Copenhagen, Denmark

## Abstract

**Background and purpose:**

Dislocation is a severe complication following total hip arthroplasty (THA). Hip precautions have been recommended in the initial postoperative period but evidence supporting this practice is limited. We therefore conducted a population-based study to evaluate the association between discontinuing recommending postoperative hip precautions and the risk of early dislocation.

**Methods:**

This is a cohort study with data from the Danish Hip Arthroplasty Register and the Danish National Patient Register. We included patients who underwent primary THA for osteoarthritis in 2004–2019 in public hospitals in the Capital Region of Denmark. The cohort was divided into the hip precautions group, comprising patients operated on between 2004 and 2009, and the no-precautions group operated on between 2014 and 2019. The primary outcome was the difference in the absolute risk of dislocation within 3 months post-surgery. The secondary outcome assessed the same risk within 2 years. We evaluated the difference in absolute risk using absolute risk regression (ARR).

**Results:**

The cumulative incidence of dislocation within 3 months was 2.9% (confidence interval [CI] 2.5–3.3) in the hip precautions group and 3.5% (CI 3.1–3.9) in the no-precautions group. The risk of dislocation was higher in the no-precautions group but failed to reach statistical significance in the crude (ARR 1.2, CI 0.9–1.6) and multivariate model (ARR 1.4, CI 0.9–2.2).

**Conclusion:**

We found a higher but statistically insignificant increase in the risk of early dislocation in the no-precautions group. The lack of significance in the association may be explained by the increased use of 36-mm femoral heads after the guideline revision.

Dislocation following total hip arthroplasty (THA) is the primary reason for revision surgery, accounting for about 20% of revisions [1,2]. Notably, nearly half of the patients with dislocations require revision within 2 years, compared with less than 2% without dislocations [3]. Furthermore, patients revised for recurrent dislocations are at increased risk of further dislocations and revisions, leading to significant health-economic costs [1,4]. Reducing dislocation risk is therefore essential. Traditional postoperative guidelines recommended limiting hip flexion, adduction, and internal rotation [5]. These guidelines lacked robust clinical evidence, despite being biomechanically meaningful [6-8]. Until June 2012, the Capital Region of Denmark’s postoperative guidelines following THA mandated hip precautions. However, studies indicated no increased dislocation rates in THA using anterior/anterolateral approaches without these precautions [9-11]. Consequently, in June 2012, the guidelines were revised to eliminate the hip precaution recommendation. Using this change in our guidelines for precautions in the Capital Region of Denmark, we performed a study with data from the Danish Hip Arthroplasty Register (DHR) and the Danish National Patient Register (DNPR), which covers all public hospitals in the Capital Region. We aimed to determine the association between the removal of hip precautions from regional postoperative guidelines and the risk of early postoperative dislocation.

## Methods

### Study design and setting

The study is reported in accordance with the RECORD guidelines [12]. The current study is a population-based cohort study drawing on prospectively collected data from the DHR and the DNPR. The DHR is a register that collects data on THAs and their subsequent revisions [13]. As of 2021, the DHR has a completeness rate of 97% for primary THAs and 95% for revisions [2]. The DNPR is an administrative database that captures all hospital contacts, including the discharge diagnoses for each contact [14]. All Danish inhabitants possess a unique 10-digit civil registration number, which facilitates linkage across administrative databases.

### Study population

The cohort was sourced from the DHR based on the following criteria: primary/idiopathic osteoarthritis (OA) diagnosis in patients who underwent THA at public hospitals in the Capital Region of Denmark from January 1, 2004, to December 31, 2019, and resided in Denmark at the time of surgery. Denmark is administratively divided into 5 healthcare regions. The study is limited only to patients from the Capital Region, as this is where the guideline was relevant. Moreover, inclusion was limited to surgeries with the posterior approach, representing over 97% of THAs in Denmark [4], and to patients with femoral head sizes of 28, 32, or 36 mm, the most commonly used sizes. We excluded patients treated at Rigshospitalet, where postoperative hip precautions were advised, patients under 40 years and patients undergoing revision surgery, patients receiving constrained liners or metal-on-metal implants, had unspecified laterality or surgical approach, or received reverse-hybrid cemented prostheses. The follow-up period was 2 years, with censoring at the first occurrence of dislocation, implant removal, death, or emigration. We considered only the first THA for patients with bilateral surgery.

### Outcomes and variables

The Capital Region of Copenhagen previously endorsed specific hip precautions advising against hip flexion exceeding 90°, adduction beyond the midline, and cautioned against internal rotation for the initial 12 weeks after the surgery. A revision of the guideline on June 29, 2012 eliminated these precautions, advocating instead for patient-guided mobilization within individual comfort zones. Dislocations were identified in the DNPR using a validated algorithm with a sensitivity of 91% and a positive predictive value of 93% [15] (Case definitions, see Appendix). The primary outcome assessed the difference in the absolute risk of dislocation within 3 months between the hip precautions and no-precautions groups. The secondary outcome evaluated the same parameter within 2 years. We retrieved demographic and surgical data, including age, sex, year of surgery, and implant specifics, from the DHR. The comorbidities dementia, history of alcohol abuse, lumbar spinal fusion, neurological motor dysfunction, and the outcome dislocation were identified through the DNPR. The study population is stratified in 2 cohorts: the hip precautions group and the no-precautions group. Some hospitals in the Capital Region abandoned hip precautions as early as 2010. For this reason, we designated patients operated on from January 1, 2004, to December 31, 2009 as the hip precautions group, marking the last known universal recommendation of hip precautions across the Capital Region. We defined the no-precautions group as patients operated on from January 1, 2014 to December 31, 2019, to ensure guideline implementation in clinical practice.

### Statistics

We report categorical variables as frequencies and percentages and continuous variables as means and standard deviations (SD). In compliance with restrictions on use of register data, we do not present results when numbers are ≤ 3. The χ2-test was used to evaluate differences in categorical variables while Welch’s t-test was used on continuous variables. Competing risk analysis with the Aalen–Johnson estimator was performed to estimate the cumulative incidence of dislocation with 95% confidence intervals (CI) up to 2 years, considering implant removal and death as competing risks. Absolute risk regression (ARR) was used to estimate the difference in dislocation risk in the no-precautions group compared with the hip precautions group [16]. This was assessed with 3 models: (i) crude estimate, (ii) adjusted for femoral head size alone, and (iii) adjusted for femoral head size, age, sex, fixation, and the comorbidities dementia, lumbar spinal fusion, alcohol abuse, and neurological motor dysfunction. We did not include body mass index (BMI) in our analysis as data on height and weight was missing for 75% of patients, with a majority missing for patients undergoing surgery in the early period. A sensitivity analysis was performed where the cut-off time for group designation was set to the date of practice change in departments that abandoned hip precautions before June 2012, or June 29, 2012 for those departments where the exact date was unknown. The detailed results from the sensitivity analysis are presented in Table 5 (see Appendix). P < 0.05 is considered significant. All data analyses were conducted in R version 4.3.1 (R Foundation for Statistical Computing, Vienna, Austria) [17]. Competing risk analysis was performed with the Prodlim package [18], while ARR was conducted with the riskRegression package [19].

### Ethics, registration, data sharing, funding, AI use, and disclosures

The Data Protection Agency of the Capital Region of Denmark approved this study (P-2022-717). Sharing of raw data from this study is not possible. Ethical approval of register studies is not required in Denmark. All data was handled via an encrypted server hosted by Statistics Denmark, where data was anonymized. Observations representing ≤ 3 individuals are not allowed to be reported due to restrictions on the use of register data. Rigshospitalet’s Research Fund (in Danish: Rigshospitalets Forskningspulje) provided funding for the study with a grant to cover the salary of 1 PhD student (AI). Kong Christian den Tiendes Fond has supported this study with a grant of DKK 50,000 for statistical assistance. The authors have no conflicts of interest to declare. The authors disclose that ChatGPT, GPT-4 (OpenAI) has been used to edit spelling and grammar as well as improving the readability and language of this manuscript. Complete disclosure of interest forms according to ICMJE are available on the article page, doi: 10.2340/17453674.2024.41168

## Results

### Baseline data

5,769 patients were classified into the hip precautions group, while 9,030 patients were included in the no-precautions group (Figure). Mean ages were 71 (SD 9.3) and 70 (SD 9.7) years, respectively, with females constituting 66% and 62%. The use of uncemented prostheses increased from 54% to 85% in the no-precautions group. Dementia, history of alcohol abuse, neuromuscular dysfunction, and previous lumbar spinal fusion was more prevalent in the no-precautions group (Table 1). There was a shift in femoral head sizes used. The most common size was 28 mm, used in 67% in the hip precautions group, while 23% received 32-mm heads and only 10% received 36-mm heads. In contrast, 73% received 36-mm heads in the no-precautions group, while 26% received 32-mm heads, and only 85 patients received 28-mm heads (Table 1). In patients with comorbidities, there was a trend towards use of 36-mm heads in the no-precautions group, compared with 28-mm and 32-mm heads in the hip precautions group (Table 2, see Appendix).

**Table 1:** Baseline characteristics for patients in the 2 groups. Results are presented as mean (standard deviation) for age as a continuous variable and number (%) for the remaining variables

Factor	Hip precautions n = 5,769	No precautions n = 9,030	P value
Age	71 (9.3)	70 (9.7)	<0.001
Age group			<0.001
< 65 years	1,474 (26)	2,500 (28)	
65–75 years	2,247 (39)	3,686 (41)	
> 75 years	2,048 (36)	2,844 (32)	
Alive after 2 years	5,541 (96)	8,801 (98)	<0.001
Sex			<0.001
Female	3,828 (66)	5,565 (62)	
Male	1,968 (34)	3,465 (38)	
Fixation			<0.001
Cemented	1,101 (19)	480 (5)	
Uncemented	3,115 (54)	7,680 (85)	
Hybrid	1,553 (27)	870 (10)	
Femoral head			<0.001
28 mm	3,842 (67)	85 (1)	
32 mm	1,347 (23)	2,335 (26)	
36 mm	580 (10)	6,610 (73)	
Comorbidities			
Dementia	36 (0.6)	33 (0.4)	0.03
Lumbar fusion	62 (1.1)	134 (1.5)	0.04
Alcohol abuse	70 (1.2)	175 (1.9)	0.001
NMD	39 (0.7)	114 (1.3)	0.001

NMD = neurologic motor dysfunction.

**Table 2:** Distribution of femoral head size by each comorbidity. Results are presented as the number (%) receiving a femoral head of either 28 and 32 mm or 36 mm of the total number of patients with each comorbidity stratified by group

Comorbidity	Hip precautions		No precautions	
	28 and 32 mm	36 mm	28 and 32 mm	36 mm
Dementia	NP	NP	13 (40)	20 (60)
Lumbar fusion	56 (90)	6 (10)	40 (30)	94 (70)
Alcohol abuse	61 (87)	9 (13)	43 (25)	132 (75)
NMD	NP	NP	44 (39)	70 (61)

NP = Not presented due to restrictions on use of register data.

NMD = neurologic motor dysfunction

**Figure:**
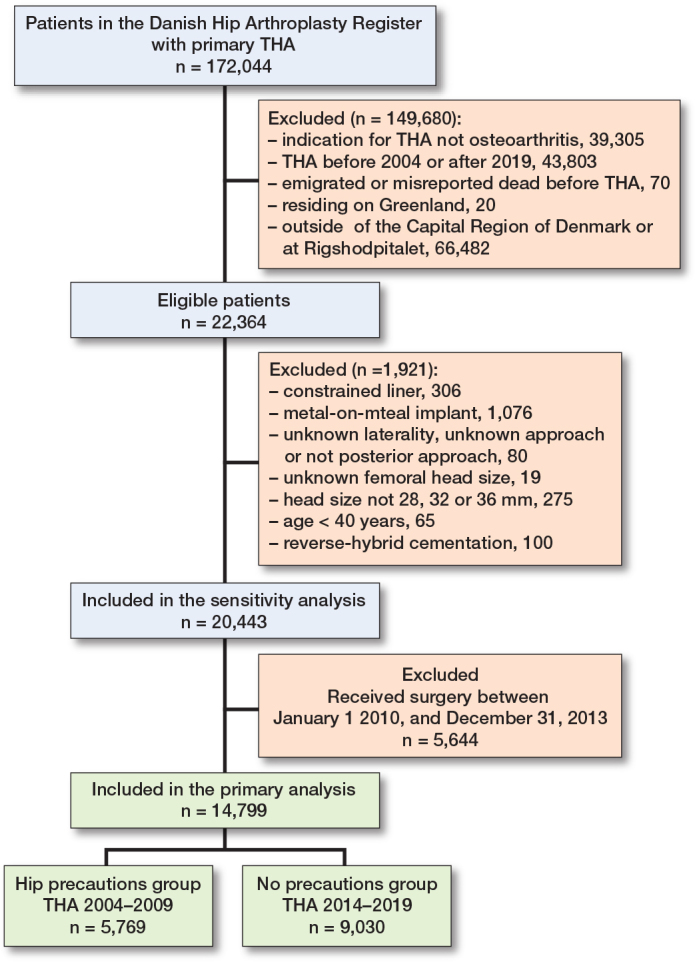
Flowchart of the study inclusion process.

### Cumulative incidence of dislocation

The cumulative incidence of dislocation was 2.9% (CI 2.5–3.3) at 3 months and 5.5% (CI 4.9–6.0) at 2 years for the hip precautions group and 3.5% (CI 3.1–3.9) and 5.0% (CI 4.5–5.4), respectively, in the no-precautions group (Table 3). The highest dislocation rates were in patients with 28-mm heads: 3.0% (CI 2.5–6.0) and 5.8% (CI 5.0–6.5) in the hip precautions group, and 5.9% (CI 0.9–10.9) and 7.1% (CI 1.6–12.5) in the no-precautions group at 3 months and 2 years, respectively. For 32-mm heads, the results were 2.5% (CI 1.7–3.4) and 5.0% (CI 3.9–6.2) in the hip precautions group, and 4.9% (CI 5.0–5.8) and 6.3% (CI 5.3–7.3) in the no-precautions group. The lowest rates were for 36-mm heads: 2.8% (CI 1.4–4.1) and 4.3% (CI 2.7–6.0) in the hip precautions group, and 3.0% (CI 2.6–3.4) and 4.5% (4.0–5.0) in the no hip precautions group (Table 3).

**Table 3:** Cumulative incidence of dislocation overall and stratified by femoral head size

Femoral head Precautions		0 to 3 months			3 months to 2 years		
		At risk	Events	Cumulative incidence (CI)	At risk	Events	Cumulative incidence (CI)
Overall	Yes	5,769	166	2.9 (2.5–3.3)	5,367	73	5.5 (4.9–6.0)
	No	9,030	317	3.5 (3.1–3.9)	8,424	61	5.0 (4.5–5.4
28 mm	Yes	3,842	116	3.0 (2.5–3.6)	3,569	54	5.8 (5.0–6.5)
	No	85	≤ 5	5.9 (0.9–10.9)	76	≤ 5	7.1 (1.6–12.5)
32 mm	Yes	1,347	34	2.5 (1.7–3.4)	1,257	15	5.0 (3.9–6.2)
	No	2,335	114	4.9 (4.0–5.8)	2,129	17	6.3 (5.3–7.3)
36 mm	Yes	580	16	2.8 (1.4–4.1)	541	≤5	4.3 (2.7–6.0)
	No	6,610	198	3.0 (2.6–3.4)	6,219	44	4.5 (4.0–5.0)

### Risk of dislocation before and after implementation of the guideline change

We found a slightly increased risk of early dislocation in the no-precautions group; however, this increase failed to reach statistical significance (Table 4). The crude model showed an increase in the no-precautions group within 3 months that did not reach significance (ARR 1.2, CI 0.9–1.6), while there was no difference within 2 years (ARR 1.0, CI 0.8–1.3). A model adjusting only for femoral head size indicated a higher dislocation risk in the no-precautions group at 3 months (ARR 1.7, CI 1.1–2.6) and 2 years (ARR 1.4, CI 1.0–1.9). However, the full model, accounting for demographics, fixation, and comorbidities, showed no significant association within either 3 months (ARR 1.4, CI 0.9–2.2) or 2 years (ARR 1.2, CI 0.9–1.7) (Table 4). The sensitivity analysis, extending the study period to include 2010–2014, aligned with the primary findings (Table 5, see Appendix). The crude model’s ARR was 1.2 (CI 0.9–1.7) at 3 months and 1.1 (CI 0.9–1.4) at 2 years. The full model showed an ARR of 1.3 (CI 1.0–1.9; P = 0.08) at 3 months and 1.2 (CI 0.9–1.5) at 2 years.

**Table 4:** Absolute risk regression (ARR) with 95% confidence intervals (CI) for the primary outcome (3 months) and secondary outcome (2 years). Table includes the crude model, the model adjusted only for femoral head and the full model adjusted for femoral head, age, sex, fixation and history of dementia, lumbar spinal fusion, alcohol abuse, and neurologic motor dysfunction (NMD)

Model	3 months		2 years	
Factor	ARR (CI)	P value	ARR (CI)	P value
Crude model				
Hip precautions	Ref.		Ref.	
No hip precautions	1.2 (0.9–1.6)	0.3	1.0 (0.8–1.3)	1
Adjusted for femoral head alone				
Hip precautions	Ref.		Ref.	
No hip precautions	1.7 (1.1–2.6)	0.01	1.4 (1.0–1.9)	0.04
32 mm	Ref.		Ref.	
28 mm	1.1 (0.8–1.7)	0.5	1.1 (0.8–1.5)	0.5
36 mm	0.65 (0.5–0.8)	< 0.001	0.7 (0.6–0.8)	< 0.001
Full model				
Hip precautions	Ref.		Ref.	
No hip precautions	1.4 (0.9–2.2)	0.1	1.2 (0.9–1.7)	0.2
32 mm	Ref.		Ref	
28 mm	0.9 (0.6–1.3)	0.6	1.0 (0.8–1.4)	0.8
36 mm	0.6 (0.4–0.8)	< 0.001	0.7 (0.5–0.9)	0.002
Age 65–75	Ref.		Ref.	
Age < 65	0.8 (0.6–1.0)	0.04	0.8 (0.6–1.0)	0.02
Age > 75	1.3 (1.1–1.6)	0.002	1.3 (1.1–1.6)	0.001
Female sex	Ref.		Ref.	
Male sex	1.4 (1.1–1.7)	0.004	1.0 (0.9–1.3)	0.6
Cemented	Ref.		Ref.	
Uncemented	1.6 (1.0–2.4)	0.04	1.2 (0.9–1.7)	0.2
Hybrid cemented	1.5 (1.0–2.2)	0.08	1.1 (0.8–1.6)	0.4
Dementia	3.9 (1.9–7.9)	< 0.001	2.5 (1.3–4.8)	0.007
Lumbar fusion	1.5 (0.8–3.0)	0.3	2.3 (1.5–3.6)	< 0.001
Alcohol abuse	1.3 (0.7–2.3)	0.4	2.4 (1.6–3.7)	< 0.001
NMD	0.7 (0.3–1.7)	0.5	0.4 (0.7–2.6)	0.4

**Table 5:** Sensitivity analysis where the cut-off period was extended to encompass the years 2010–2014 (see Methods section). Table presents the sensitivity analysis for the absolute risk regression (ARR) with 95% confidence intervals (CI) for the primary outcome (3 months) and secondary outcome (2 years). Table includes the crude model, the model adjusted only for femoral head, and the full model adjusted for femoral head, age, sex, fixation, and history of dementia, lumbar spinal fusion, alcohol abuse, and neurologic motor dysfunction (NMD)

Model	3 months		2 years	
Factor	ARR (CI)	P value	ARR (CI)	P value
Crude model				
Hip precautions	Ref.		Ref.	
No hip precautions	1.2 (0.9–1.7)	0.2	1.1 (0.9–1.4)	0.4
Adjusted for femoral head alone				
Hip precautions	Ref.		Ref.	
No hip precautions	1.5 (1.1–2.1)	0.01	1.4 (1.1–1.7)	0.02
32 mm	Ref.		Ref.	
28 mm	1.0 (0.8–1.4)	0.8	1.1 (0.9–1.4)	0.4
36 mm	0.6 (0.6–0.9)	< 0.001	0.7 (0.6–0.8)	< 0.001
Full model				
Hip precautions	Ref.		Ref.	
No hip precautions	1.3 (1.0–1.9)	0.08	1.2 (0.9–1.5)	0.2
32 mm	Ref.		Ref.	
28 mm	0.9 (0.7–1.3)	0.6	1.0 (0.8–1.3)	1
36 mm	0.6 (0.5–0.8)	< 0.001	0.7 (0.6–0.8)	< 0.001
Age 65–75	Ref.		Ref.	
Age < 65	0.7 (0.6–0.9)	0.004	0.8 (0.6–0.9)	< 0.001
Age > 75	1.5 (1.2–1.7)	< 0.001	1.4 (1.2–1.6)	< 0.001
Female sex	Ref.		Ref.	
Male sex	1.3 (1.1–1.5)	< 0 .001	1.0 (0.9–1.2)	0.7
Cemented	Ref.		Ref.	
Uncemented	1.9 (1.3–2.7)	< 0.001	1.5 (1.1–1.9)	0.007
Hybrid cemented	1.6 (1.1–2.4)	0.008	1.3 (1.0–1.7)	0.1
Dementia	3.0 (1.5–5.9)	< 0.001	2.5 (1.4–4.3)	< 0.001
Lumbar fusion	1.6 (0.9–2.9)	0.09	2.2 (1.4–3.3)	< 0.001
Alcohol abuse	2.0 (1.3–3.0)	0.002	2.8 (1.9–3.9)	< 0.001
NMD	1.3 (0.8–2.4)	0.3	1.7 (1.1–2.7)	< 0.001

## Discussion

We aimed to assess whether omitting the recommendation for hip precautions from the regional guideline on postoperative mobilization following THA increased the risk of early dislocation. Contrary to our hypothesis, the study did not find a clear association between discontinuing the recommendation for hip precautions and the risk of early dislocation within 3 months or 2 years. Secondary analysis accounting solely for femoral head size showed an increased dislocation risk in the no-precautions group, suggesting a protective effect of hip precautions in patients receiving 28-mm and 32-mm femoral heads. However, after also accounting for age, sex, fixation, and comorbidities in the full model, the removal of hip precautions from the guideline again failed to reach significance for an increased dislocation risk. Our findings imply that the elevated dislocation risk in certain patients is mitigated by using larger femoral heads, thus questioning the necessity of hip precautions in patients receiving THA with femoral heads ≥36 mm.

### Comparison with current literature

Hip precautions have traditionally been advocated in the early phase after THA to mitigate early dislocation risk [6]. However, the scientific basis for persistently recommending these precautions has been questionable [7,8], with studies on patients receiving THA with the anterior/anterolateral approach finding no benefits of hip precautions [9-11]. In Denmark, the posterior approach, which is associated with a higher dislocation risk [19], is predominantly used [2], leading to suggestions that hip precautions could be beneficial in these cases. Existing clinical studies have not demonstrated any advantage of hip precautions in decreasing early dislocation risk for the posterior approach [20]. However, a common limitation across these studies is their design; most are single-center observational studies or small trials, which are underpowered due to the limited number of participants. Our population-based analysis, which exclusively included patients undergoing the posterior approach, supports previous findings that show no heightened risk when recommendations for hip precautions are omitted. Our findings are consistent with those from a similar study using a national administrative dataset from England [21]. Nonetheless, it is crucial to consider our results within the context of a rising proportion of patients receiving THA with 36-mm femoral heads, alongside concurrent advancements and heightened awareness in surgical techniques. Over time, there has been greater attention to factors known to decrease dislocation risk, such as cup positioning and meticulous capsular repair. These factors might have been more prevalent in the no-precautions group, who underwent surgery later compared with the hip precautions group [22,23].

### High-risk patients and large-diameter femoral heads

We observed a higher proportion of patients with risk factors for dislocation in the no-precautions group, indicating a shift in the patient demographics of OA undergoing THA. Notably, there was an increased prevalence of neurologic motor dysfunction, prior lumbar spinal fusion surgery, and uncemented THA in the no-precautions group, which are known risk factors for dislocation [4,19]. These differences likely influenced the results of our ARR model that considers only femoral head size, as the disparities were not significant once we adjusted for comorbidities, fixation, and femoral head size. In the no-precautions group, a majority were fitted with 36-mm femoral heads, contrasting with a minority in the hip precautions group. Large-diameter femoral heads correlate with lower dislocation rates among high-risk patients [24,25]. Notably, 36-mm femoral heads are recognized for reducing dislocation compared with their 32-mm counterparts, with added efficacy in patients undergoing THA with the posterior approach [24,25]. Consequently, the potential negative impacts linked to discontinuing hip precautions are likely offset by the augmented adoption of large-diameter femoral heads.

### Hip precautions and compliance

Our study uses the guideline’s inclusion of hip precautions as proxy for effectiveness of precautions. Our design is not able to assess the direct impact of the implementation of hip precautions, patient adherence, or healthcare practitioners’ compliance with guideline recommendations and subsequent omissions: 1 study evaluating hip precautions revealed that about 25% of patients in the hip precautions group did not adhere, while 20% in the no-precautions group followed some precautions unintentionally [26]. Additionally, 1 study on compliance found only 23% adhered to precautions after 6 weeks, despite 86% believing they could recall them [27], while another reported a mere 6% maintained restrictions for the full recommended timespan of 12 weeks [28]. Qualitative research shows challenges among healthcare providers, including doctors and nurses, in adapting to new protocols [29,30]. Difficulty in breaking from established routines leads to occasional reversion to old practices of recommending precautions, despite the official removal of these [29]. Communication inconsistencies also arise due to non-adherence to updated protocols by some colleagues or lack of awareness among external post-discharge therapists [29]. Furthermore, some continue to advise precautions for at-risk patients despite changes in practice guidelines [30].

### Strengths and limitations

The main strengths of our study lie in its population-based design and linkage with the DNPR, facilitating the inclusion of data on comorbidities and hip dislocations. The latter was identified using a validated algorithm with an excellent sensitivity and positive predictive value [15]. Our study has certain limitations that need to be addressed. Despite an 18-month period for the adoption of the new guideline, the risk of incomplete implementation remains. We were unable to assess individual compliance, the degree of adherence to precautions, or the other pitfalls mentioned in the previous paragraph, as neither DHR nor DNPR records such information. Additionally, group allocation was based on a time cut-off, not randomization. Our analysis assumes that the guideline change was the primary factor influencing early dislocation risk. To mitigate other variables, we incorporated patient, surgical, and implant characteristics into an adjusted model. Nevertheless, the potential for residual confounding cannot be discounted, as there are other factors relevant for dislocation that we could not account for, either due to a lack of reporting in the databases such as whether or not capsular repair was performed, or due to a significant amount of missing data such as for BMI. It is worthwhile noting that we consistently found a higher dislocation risk in the no-precautions group even though these findings generally failed to reach significance, which is why our study does not clearly exclude the possibility that hip precautions could be of importance for certain patients.

### Conclusion

We did not find a clear association between discontinuing the recommendation for hip precautions and the risk of early dislocation within 3 months or 2 years. There may be a potential role for hip precautions in patients receiving 28-mm or 32-mm femoral heads and in certain high-risk patients but not in patients with ≥ 36 mm heads.

## Notes

AI: design of the study, data analysis, interpretation of the results, writing of the original draft, critical revisions, and funding acquisition. MSS: design of the study, supervision, interpretation of the results, and critical revisions of the manuscript. MW-J: data analysis, supervision, interpretation of the results, and critical revisions of the manuscript. SO: design of the study, interpretation of the results, and critical revisions of the manuscript. MMP: conceptualization and design of the study, interpretation of the results, critical revisions of the manuscript, and funding acquisition.

Handling co-editors: Bart A. Swierstra and Philippe Wagner

*Acta* thanks Ian Harris and Matthijs P Somford for help with peer review of this manuscript.

## Appendix

### Primary total hip arthroplasty due to primary osteoarthritis

The patient cohort was sourced from the Danish Hip Arthroplasty Register (DHR), which exclusively records total hip arthroplasties (THA). To identify patients who underwent primary THA with osteoarthritis as the underlying condition, data was filtered using the criteria “Primary (idiopathic) arthrosis” under the “Underlying condition” column and “Primary surgery” under the “Surgery group” column, denoted by OGRUNDL = 1 & opgrp = “Primær”.

### Dislocation

Dislocations were identified in the Danish National Patient Register (DNPR) using ICD-10 codes and/or NOMESCO procedure codes and classified as either true dislocations or probable dislocations as defined by Hermansen et al. [15]. The dislocation rate reported in this article is the first occurrence of either a true or a probable dislocation:

- True dislocation
  Diagnosis code T840(A) (Mechanical complication of internal joint prosthesis) and procedure code NHF20 (Closed reduction of dislocation prosthesis of hip joint) and a known laterality equal to the laterality of the THA
- Probable dislocation
  Minimum 1 of the following codes either with a known laterality equal to the laterality of the THA or an unknown laterality:
    S730 Dislocation of hip
    NFH00 Reduction of dislocation of hip joint - Closed
    NFH02 Reduction of dislocation of hip joint - Open
    NFH20 Reduction of dislocation prosthesis of hip joint - Closed
    NFH21 Reduction of dislocation prosthesis of hip joint - Arthroscopic
    NFH22 Reduction of dislocation prosthesis of hip joint - Open

### Comorbidities

The presence of a comorbidity was defined as at least one in-hospital or outpatient administrative contact preceding the date of the primary surgery with one of the following ICD-10 diagnosis or NOMESCO procedure code:

- Dementia
  F00 Dementia in Alzheimer disease
  F01 Vascular dementia
  F02 Dementia in other diseases classified elsewhere
  F03 Unspecified dementia
  G30 Alzheimer disease
- Alcohol abuse
  F10.1 Mental and behavioral disorders due to use of alcohol – Harmful use
  F10.2 Mental and behavioral disorders due to use of alcohol – Dependence syndrome
- Neurologic motor dysfunction
  G10 Huntington disease
  G11 Hereditary ataxia
  G12 Spinal muscular atrophy and related syndromes
  G13 Systemic atrophies primarily affecting central nervous system in diseases classified elsewhere
  G14 Post-polio syndrome
  G20 Parkinson disease
  G21 Secondary Parkinsonism
  G22 Parkinsonism in diseases classified elsewhere
  G23 Other degenerative diseases of basal ganglia
  G25 Other extrapyramidal and movement disorders
  G26 Extrapyramidal and movement disorders in diseases classified elsewhere
  G80 Cerebral palsy
  G81 Hemiplegia
  G82 Paraplegia and tetraplegia
  G83 Other paralytic syndromes
- Spinal fusion of the lumbo-sacral spine
  NAG33 Interbody fusion of spine without fixation: Thoraco-lumbar spine
  NAG34 Interbody fusion of spine without fixation: Lumbar spine
  NAG35 Interbody fusion of spine without fixation: Cervico-thoraco-lumbar spine
  NAG36 Interbody fusion of spine without fixation: Cervico-thoraco-lumbar spine
  NAG43 Interbody fusion of spine with internal fixation: Thoraco-lumbar spine
  NAG44 Interbody fusion of spine with internal fixation: Lumbar spine
  NAG45 Interbody fusion of spine with internal fixation: Cervico-thoraco-lumbar spine
  NAG46 Interbody fusion of spine with internal fixation: Lumbo-sacral spine
  NAG63 Interlaminary fusion of spine without fixation: Thoraco-lumbar spine
  NAG64 Interlaminary fusion of spine without fixation: Lumbar spine
  NAG65 Interlaminary fusion of spine without fixation: Cervico-thoraco-lumbar spine
  NAG66 Interlaminary fusion of spine without fixation: Lumbo-sacral spine
  NAG73 Interlaminary fusion of spine with fixation: Thoraco-lumbar spine
  NAG74 Interlaminary fusion of spine with fixation: Lumbar spine
  NAG75 Interlaminary fusion of spine with fixation: Cervico-thoraco-lumbar spine
  NAG76 Interlaminary fusion of spine with fixation: Lumbo-sacral spine
  NAG83 Unilateral intertransverse fusion of spine: Thoraco-lumbar spine
  NAG84 Unilateral intertransverse fusion of spine: Lumbar spine
  NAG85 Unilateral intertransverse fusion of spine: Cervico-thoraco-lumbar spine
  NAG86 Unilateral intertransverse fusion of spine: Lumbo-sacral spine
  NAT13 Anterior traction of spine using internal correctional instrument: Thoraco-lumbar spine
  NAT14 Anterior traction of spine using internal correctional instrument: Lumbar spine
  NAT15 Anterior traction of spine using internal correctional instrument: Cervico-thoraco-lumbar spine
  NAT16 Anterior traction of spine using internal correctional instrument: Lumbo-sacral spine
  NAT23 Posterior traction of spine using internal correctional instrument: Thoraco-lumbar spine
  NAT24 Posterior traction of spine using internal correctional instrument: Lumbar spine
  NAT25 Posterior traction of spine using internal correctional instrument: Cervico-thoraco-lumbar spine
  NAT26 Posterior traction of spine using internal correctional instrument: Lumbo-sacral spine
